# Wide awake local anaesthesia no tourniquet technique (WALANT)

**DOI:** 10.1186/1753-6561-9-S3-A81

**Published:** 2015-05-19

**Authors:** Donald Lalonde

**Affiliations:** 1 Department of Plastic and Reconstructive Surgery, Dalhousie University, Saint John, New Brunswick, Canada, E2K 1J5

## 

Until recently most hand surgeries was performed with a tourniquet to provide better visibility. The discomfort of the tourniquet is very unpleasant and unnecessary for patients. To avoid this, we have traditionally relied on an anesthesiologist to give sedation, brachial plexus or Bier block, or general anesthesia.

A good alternative to traditional tourniquet hand surgery is to use only 2 medications; lidocaine for anesthesia and epinephrine for hemostasis.

Lidocaine and epinephrine are likely two of the safest and most widely tested drugs known to mankind. Billions of doses of these two medications have been injected into people for simple dental procedures in dental offices since 1950 with no preoperative testing, no monitoring, no intravenous insertion, and very few adverse events. Epinephrine in the finger is now known to be safe (*Lalonde DH*, *Martin A. Epinephrine in local anesthesia in finger and hand surgery: The case for wide-awake anesthesia. J Am Acad Orthop Surg; 2013;21*(*8*)*:443*)*.* Finger necrosis blamed on epinephrine before the1950s when the epinephrine myth was created is now known to have been caused by procaine.

Like dental procedures, wide awake hand surgery can be performed with no preoperative testing, no intravenous insertion, and no monitoring. The patient simply gets up and goes home after the procedure.

## Advantages for the patient

• No cost for time out of work (or need for getting a baby sitter) to go get preoperative testing for sedation

• Hand surgery under local is not expensive. Many patients could afford it if they did not have to pay the large expenses associated with sedation

• No unnecessary preoperative needles for test, EKG, chest Xray, anesthesia consultations, etc

• No unnecessary intravenous insertion

• Less time at the hospital for the procedure as there is no recovery time.

• No need for the patient to have someone be with him the evening of the surgery as is often mandated after sedation

• Get to talk to their surgeon during the surgery for post-operative advice on how to look after the hand, time out of work, etc.

• Get to see repaired structures working during the surgery after loss of function such as tendon laceration or Dupuytren’s contracture. This visual memory helps motivate the patient in post-operative therapy and recovery.

• No need to endure the unnecessary tourniquet, even for 5 minutes. We tell all our trainees that they need to put a tourniquet on their own arm or forearm for 5 minutes before they ever say something like: “Patients tolerate it well” which really means: “They let me do it even though it hurt”

• No need to fast or change medication schedules; particularly helpful in diabetics

• Patients with sore elbows, shoulders or backs can position themselves comfortably for the hand and elbow surgery (cubital tunnel release) as there is no tourniquet or anesthesiology equipment in the way

• They can just sit up and leave right after the surgery without having to recover from sedation or unnecessary opiates.

## Advantages for the surgeon

• Only one nurse required to perform most simple hand operations such as carpal tunnel release. This greatly increases efficiency, reduces costs, and increases productivity for the surgeon. I can do 2 times the number of cases in the same amount of time at ¼ the cost of traditional tourniquet/sedation hand surgery

• The surgeon can move simple operations like carpal tunnel and trigger finger out of the operating room and perform them in the clinic or office with field sterility for greatly improved turnover time and patient convenience

• Get to educate the patient during the surgery for better outcomes and fewer complications

• Get to make adjustments on repaired tendons and bones after seeing active movement in comfortable cooperative patients before the skin is closed to make sure everything is working well to improve results

• Do not have to look after in patients who are admitted after hand surgery because of sedation

• Patients with multiple medical problems can be looked after safely and easily as their conditions are not affected by sedation. They walk in, have their hand surgery, and then get up and go home just as they do for a dental filling in a dental office.

• Get to set proper tension on tendon transfers before closing the skin

• Get to see what is happening with active movement during the surgery in complex reconstructive hand surgery cases to improve results.

## Why no sedation?

• Increased safety: The safest sedation is no sedation

• No need for sedation: The only medical reasons patients needed sedation in the past for hand surgery were 1) to tolerate the pain of the tourniquet and 2) to tolerate the pain of injection of the local anesthesia). Those reasons no longer exist as there are now ways to inject local anesthesia with minimal pain.

• Major decrease in cost. Hand surgery is not expensive. Safe sedation is expensive. Poor patients can afford hand surgery if the sedation component is eliminated.

• Patients can receive education from their surgeon and therapist during the surgery as they have no anamnestic drugs to impair their memory and learning ability (see chapter 7 on patient education during surgery)

• Pain free unsedated cooperative patients can take reconstructed parts through a full range of active movement during the surgery and remember how well the reconstructed hand functions. The surgeon can make changes during the procedure to improve the outcome. These functions are particularly important in setting the tension in tendon transfer, making sure there is no gapping and that the tendons fit through the pulleys after flexor tendon repair, and see how stable the fixation is with intraoperative active movement after fracture reduction.

• Some patients become uninhibited and harder to manage with small amounts of sedation and end up needing general anesthesia with all its risks.

• Sedated patients will not remember intraoperative teaching from their surgeon and miss this excellent communication opportunity.

